# Supplementary material for: Complexome profiling on the Chlamydomonas lpa2 mutant reveals insights into PSII biogenesis and new PSII associated proteins
Source: J Exp Bot. 2021 Aug 26;73(1):245–62. doi: 10.1093/jxb/erab390 (PMC8730698; doi:10.1093/jxb/erab390)
Supplement: erab390_suppl_Supplementary_Dataset_S1 [file erab390_suppl_supplementary_dataset_s1.zip › Supplemental Dataset 1 - Excel List and all profiles/plots/LEU1S_Cre06.g252650.html]

Trivial name: LEU1S  
  
Euclidean distance: 3752.85  
Mean Intensity (WT): 162.65  
Mean Intensity (Mut): 266.17  
Distance: 14.10  
  
MapMan: secondary metabolism.sulfur-containing.glucosinolates.synthesis.aliphatic.methylthioalkylmalate isomerase small subunit (MAM-IS);amino acid metabolism.degradation.branched chain group.leucine  
  
p value of intensity sums Welch test: 0.5137
